# A Novel Compression Garment With a Dynamic External Lymphatic Drainage System: A Proof of Concept Study

**DOI:** 10.1093/asjof/ojag010

**Published:** 2026-01-28

**Authors:** Ali Mojallal, Fabien Boucher, Zmorda EI Messioui, Léa Clément, Haïzam Oubari

**Affiliations:** From the Department of Plastic, Reconstructive and Aesthetic Surgery, Hôpital de la Croix-Rousse, CHU de Lyon, Hospices Civils de Lyon, Lyon, France; From the Department of Plastic, Reconstructive and Aesthetic Surgery, Hôpital de la Croix-Rousse, CHU de Lyon, Hospices Civils de Lyon, Lyon, France; From the Department of Plastic, Reconstructive and Aesthetic Surgery, Hôpital de la Croix-Rousse, CHU de Lyon, Hospices Civils de Lyon, Lyon, France; From the Department of Plastic, Reconstructive and Aesthetic Surgery, Hôpital de la Croix-Rousse, CHU de Lyon, Hospices Civils de Lyon, Lyon, France; From the Department of Plastic, Reconstructive and Aesthetic Surgery, Hôpital de la Croix-Rousse, CHU de Lyon, Hospices Civils de Lyon, Lyon, France

## Abstract

**Background:**

Postoperative compression has been widely used for over half a century to reduce lymphatic stasis, pain, and edema, while promoting healing and optimal skin redraping. However, most commercially available garments share similar designs, with little innovation in recent decades.

**Objectives:**

To present and evaluate a novel compression garment integrating an external lymphatic drainage system designed to enhance postoperative recovery.

**Methods:**

Based on anatomical lymphatic pathways, we developed and patented a novel compression garment (Drain Lipo Panty®, DLP) incorporating skin-adherent silicone strips aligned with physiological drainage lines. Prototype garments were designed with unilateral silicone integration, allowing intra-patient comparison. This prospective, single-center study included patients undergoing lipoabdominoplasty and/or lower-limb liposuction. Assessments were performed at postoperative days 0, 7, 15, 30, and 60 by an independent evaluator. Outcomes included objective edema measurements, standardized photographic analysis, and patient-reported comfort scores.

**Results:**

Fifty-two patients were included (mean follow-up: 2 months). The silicone-strip side showed faster edema resorption and improved skin recovery. In the lipoabdominoplasty group, edema reduction was significantly greater at day 30 (T1, *P* = .001; T2, *P* = .004). In the lower-limb liposuction group, mean circumference reduction was 8.68 cm with the silicone garment vs 6.40 cm with the standard garment (*P* < .001).

**Conclusions:**

This novel compression garment with integrated silicone drainage strips demonstrated faster edema resolution, earlier ecchymosis recovery, and improved skin redraping, suggesting a beneficial role in postoperative lymphatic rehabilitation.

**Level of Evidence:**

: 3 (Therapeutic) 
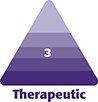

Postoperative compression therapy has been a mainstay in surgical recovery for more than half a century, with well-established benefits in reducing edema, improving patient comfort, and supporting aesthetic outcomes, particularly in procedures that involve significant disruption of lymphatic pathways such as lipoabdominoplasty and lower limb liposuction. However, despite its widespread adoption, technical innovation in compression garment design has remained limited, and persistent postoperative edema continues to be a common concern that may contribute to fibrosis, pain, delayed healing, and suboptimal contours. Notably, the most recent French National Authority for Health (HAS) guidelines for lymphatic insufficiency date back to 2010, underscoring a longstanding stagnation in evidence-based development and garment optimization.^1^

Recent advances in lymphatic imaging have renewed interest in the anatomy and dynamics of lymphatic drainage after surgery. Indocyanine green (ICG) lymphography allows real-time visualization of superficial lymphatic pathways and has been used to evaluate the effect of manual interventions on lymphatic flow.^2^ Using this technique, Suami et al^3^ identified 8 drainage regions in the lower limbs, with the inguinal and popliteal basins serving as primary outflow pathways, while additional collateral territories appear mainly under conditions of lymphatic overload or insufficiency. Cadaveric investigations have further demonstrated the organization of lower limb lymphatic drainage into 4 main lymphatic groups converging toward 3 dominant nodal basins.^4^ In the abdomen, Bassalobre et al^5^ observed postoperative shifts in lymphatic drainage patterns, with rerouting toward axillary nodes occurring in a substantial proportion of patients following abdominoplasty. Taken together, these studies suggest that lymphatic drainage pathways are both anatomically patterned and modifiable after surgical intervention, indicating that uniform, non-directed compression may not always support optimal lymphatic recovery. In parallel, emerging evidence suggests that materials with skin-adherent properties, such as silicone, may enhance the functional performance of compression garments by improving tissue contact, stimulating microcirculation, and promoting lymphatic mobilization.^6,7^

Based on these observations, we developed and patented a novel postoperative compression garment incorporating sinusoidal silicone strips aligned with lymphatic drainage pathways. These strips adhere directly to the skin and apply gentle, movement-dependent pressure intended to facilitate directed lymphatic flow. The objective of this prospective, single-center study is to evaluate the effectiveness of this novel compression garment in postoperative recovery following lipoabdominoplasty and lower limb liposuction. Using a patient-controlled, intra-individual comparison design, we assess whether the integration of anatomically aligned silicone strips improves edema resorption, skin redraping, and overall comfort compared with conventional compression garments.

## METHODS

In this monocentric prospective study, all surgical procedures were performed by the senior surgeon (Dr. Ali Mojallal). Follow ups and assessments were conducted at the Croix Rousse Hospital Plastic Surgery Department (Lyon, France) by French board-certified plastic surgeons. No Institutional Review Board approval was required for the use of the garment, as passive (ie, non-externally powered) compression garments are already in routine use at our institution, are approved for use in the European Union, and this new garment utilizes only approved clinical-grade materials. The study was conducted between January 2023 and March 2024 and follows the principles of the Declaration of Helsinki.

### Population

The study population consisted exclusively of female patients, divided into 2 groups. All patients underwent liposuction using the tumescent technique, with approximately 500 mL of saline mixed with adrenaline 1% per area, in a ratio equal to the desired liposuction volume. All procedures were performed symmetrically. The first group included those who underwent lipoabdominoplasty, which involved abdominal, lateral suprailiac, and lumbar liposuction. The second group comprised patients who underwent lower limb liposuction, targeting the trochanteric regions (hips), inner thighs, and knees, combined with a vertical cruroplasty-type skin resection. Patients who had both procedures were included in both groups. Inclusion criteria were female patients aged 18 and over who had undergone abdominoplasty with circumferential liposuction and/or lower limb liposuction. Participants had to be willing to follow postoperative recommendations, including wearing the assigned compression garment for the prescribed period. Only those capable of understanding and providing informed consent were included, with a focus on individuals at higher risk of postoperative lymphatic complications, such as edema. Exclusion criteria included male patients, minors, pregnant or breastfeeding women, and those unable to comply with postoperative recommendations. Patients with pre-existing lymphatic disorders that could interfere with the study results or medical contraindications to compression garment use, such as allergies to garment materials (eg, silicone allergy), were also excluded.

### Drain Lipo Panty Compression Garment

**Figure 1.**
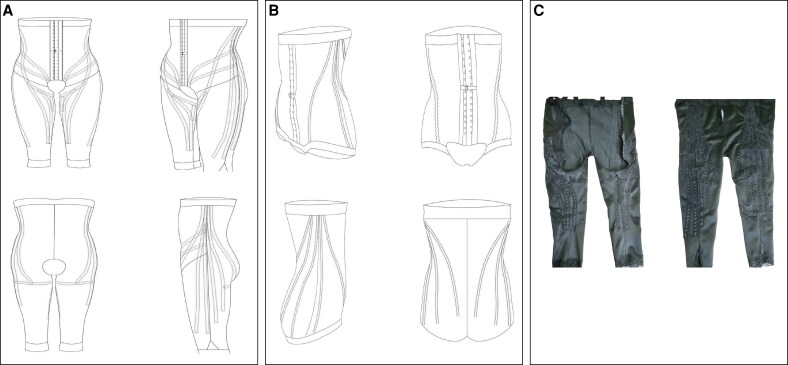
Schematic of the garment for (A) lipoabdominoplasty and lower limb liposuction, (B) abdominoplasty, and (C) clinical photograph showing the inner silicone bands.

The postoperative compression garment used in this study is specifically designed to enhance lymphatic drainage. It incorporates a patented lymphatic drainage system (Drain Lipo Panty, DLP; patent no. 227574) that adheres closely to the skin, optimizing lymphatic fluid resorption and reducing postoperative edema. The mechanism relies on the local adherence of internal sinusoidal silicone strips positioned along cutaneous lymphatic drainage pathways (Figure 1A-C). These strips create a semi-passive drainage process, activated by the patient's natural movements, without requiring an external power source. Their wave-shaped reliefs exert gentle, intermittent pressure on surrounding tissues, thereby facilitating the mobilization of lymphatic fluid toward regional lymph nodes.^8^ The goal is to enhance fluid circulation, reduce postoperative edema, and accelerate recovery.

### Study Endpoints

**Figure 2.**
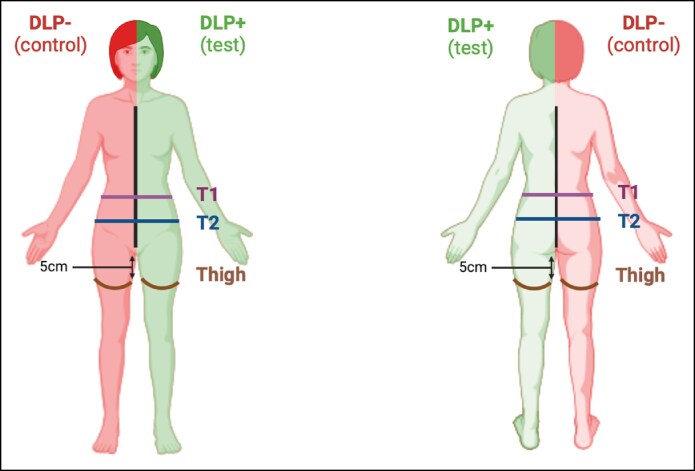
Body measurements schematic. DLP, Drain Lipo Panty.

The primary endpoint of the study was the resorption of postoperative edema, assessed through multiple measurements and was recorded preoperatively at D0 and postoperatively at D7, D15, D30, and D60 using a tape measure. The measurements were performed by authors (F.B., Z.M., L.C.) who were not involved in the surgical procedures conducted by Dr. A. Mojallal. A garment prototype was specifically developed for this study: one side with silicone strips (termed as DLP+), the contralateral side without silicone strips (conventional compression garment, termed as DLP-). Patients served as their own controls. During the preoperative consultation, a compression garment with silicone strips was fitted on either the right or left side based on availability and appropriate sizing (Video). For the lipoabdominoplasty group with circumferential liposuction, measurements were taken as follows (Figure 2):

- T1 measurement (cm) = HemiT1 measurement L/R: The distance from the midline to the line of the spines on the left or right side at the horizontal level passing through the umbilicus.
- T2 measurement (cm) = HemiT2 measurement L/R: The distance from the midline to the line of the spines on the left or right side at the horizontal level passing through the anterior superior iliac spine (EIAS).

For the lower limb liposuction group measurements were made as follows:

- Thigh circumference (cm) L/R: Measurement of the thigh circumference 5 cm below the inner root of the thigh on the left or right side.

Secondary endpoints included patient comfort, assessed using a subjective evaluation questionnaire (Supplemental Figure) completed at the end of the follow-up period, as well as the presence of ecchymosis and the degree of skin redraping, both evaluated by the physician and by analysis of standardized follow-up photographs performed by the last author (H.O.), who was blinded to the follow-up and surgical procedures.

### Statistical Analysis

The variables were recorded and analyzed using SPSS software (IBM SPSS Statistics, Version 21.0, IBM Corp., Armonk, NY). The normality of continuous quantitative variables was assessed using the Shapiro–Wilk test. Quantitative variables were expressed as mean ± standard deviation. The Student *t*-test was used to compare the means of 2 independent groups, while the paired samples *t*-test was applied to assess mean variations within the same group. All statistical tests were 2-tailed, with the significance level set at 5%.

## RESULTS

### Patient Characteristics

A total of 52 patients were included in the study: 22 underwent isolated lipoabdominoplasty, 22 underwent lower limb liposuction, and 8 underwent both procedures. The mean age was 45 years and the mean body mass index (BMI) was close to 25 kg/m² (Table 1). One patient reported a prior history of liposuction before enrollment, and no complications occurred during the postoperative course.

**Table 1.** Study Population Characteristics

Variable	Minimum	Maximum	Mean	Standard error
Age (years)	29.00	67.00	45.00	1.28
Weight (kg)	58.00	85.00	68.98	0.76
Height (cm)	156.00	181.00	166.33	.79
BMI (kg/m²)	20.50	29.20	24.96	0.26

### Resorption of Postoperative Edema

**Figure 3.**
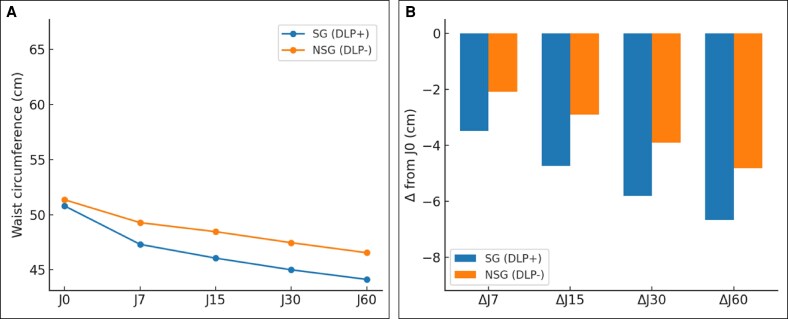
(A) T1 waist circumference analysis, with T1 mean waist circumference and (B) T1 waist circumference variation over time. *P *<* 0.05; **P *<* 0.01; ***P *<* 0.001. DLP, Drain Lipo Panty; SG.

**Figure 4.**
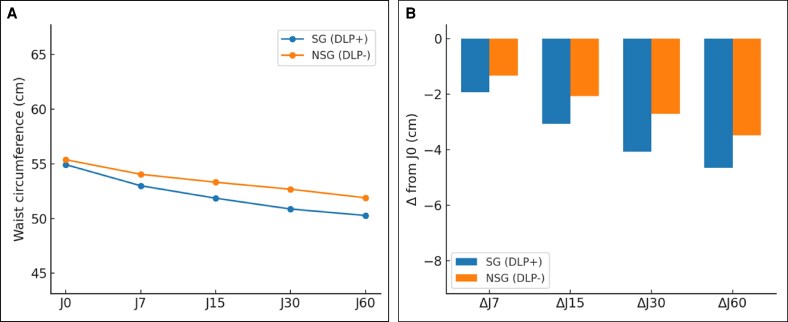
(A) T2 Waist circumference analysis, with T2 mean waist circumference and (B) T2 waist circumference variation over time. *P *<* 0.05; **P *<* 0.01; ***P *<* 0.001.

**Figure 5.**
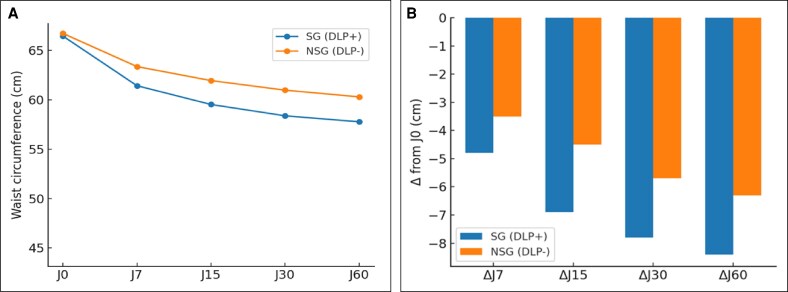
(A) Thigh waist circumference analysis with thigh mean waist circumference and (B) thigh waist circumference variation over time (B). *P *<* 0.05; **P *<* 0.01; ***P *<* 0.001.

**Figure 6.**
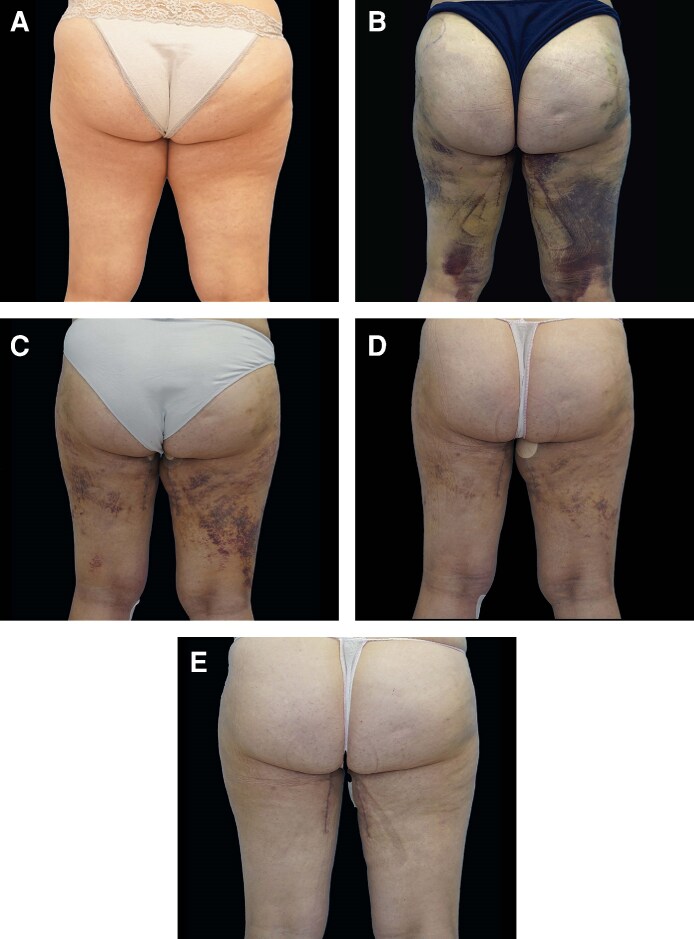
Example of a 45-year-old female patient who underwent lower-limb liposuction and medial thigh lift and wore the prototype garment for the first 2 postoperative months: continuously day and night during the first month, then during the day only for the second month. Photographs were taken (A) before surgery, (B) at POD 7, (C) POD 14, (D) POD 21, (E) and POD 60. Note the skin marks left by the silicone bands on the left side (DLP+), with the right side serving as control (DLP−).

**Figure 7.**
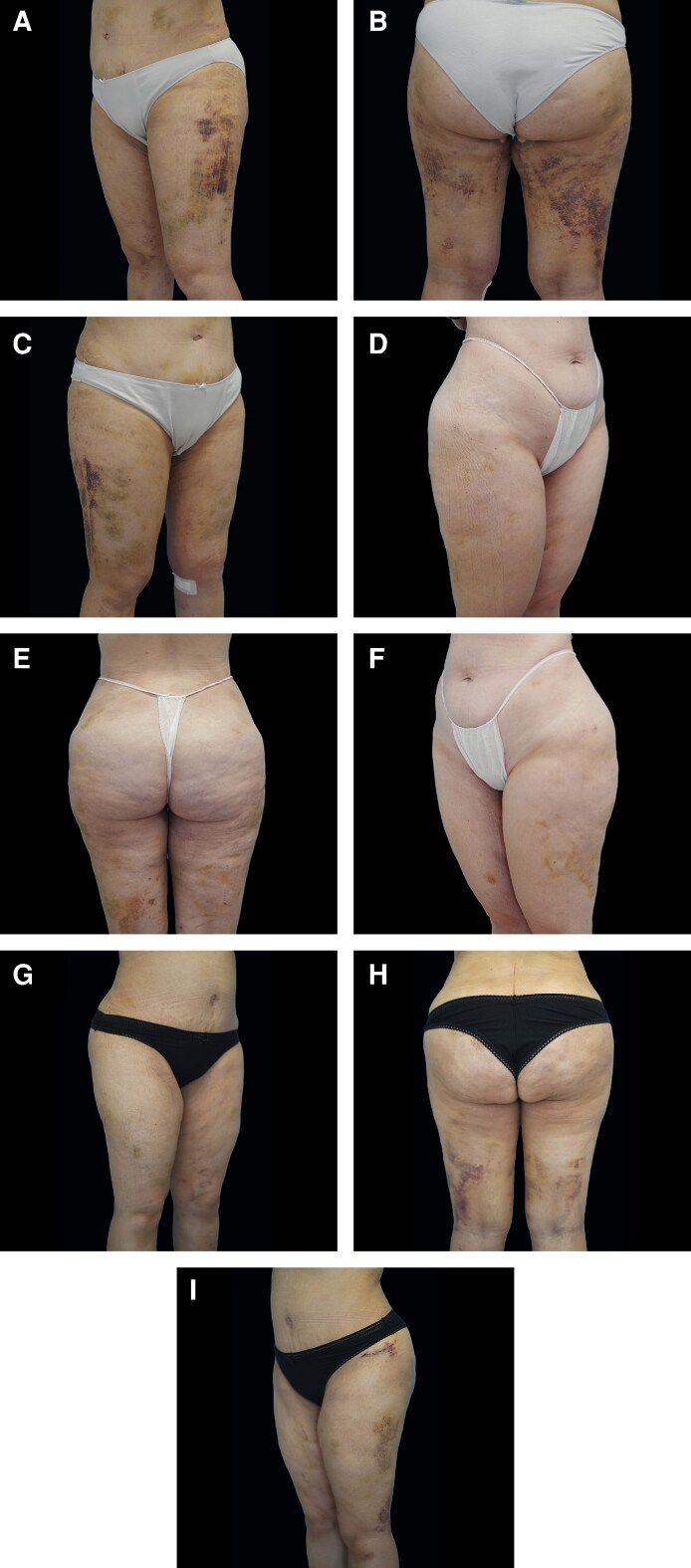
Three examples of POD 14 postoperative outcomes after flank and lower-limb liposuction in patients wearing a double-sided prototype garment, with DLP+ sides shown in panels (A, D, G) and DLP− sides in panels (C, F, I). Shown are (A-C) a 45-year-old female, (D-F) a 36-year-old female, and (G-I) a 49-year-old female.

At baseline (D0), no differences were observed between the DLP + and DLP– sides across T1, T2, and thigh measurements, confirming the comparability of groups (see Figures 3-5, “D0” measurement on the *x* axis). A progressive reduction in postoperative edema was measured at all anatomical sites, with consistently greater decreases on the DLP + side compared to the DLP– side for abdominal assessments. In the lipoabdominoplasty group, T1 waist circumference decreased from 50.8 cm at D0 to 44.1 cm at D60 with DLP+, vs 51.4 cm to 46.6 cm with DLP–, corresponding to mean variations of –6.7 cm vs −4.8 cm (*P* = .002; Figure 3). At T2, reductions reached −4.7 cm with DLP + compared to −3.5 cm with DLP− at D60 (*P* = .014; Figure 4). In the lower limb group, thigh circumference decreased from 66.5 cm to 57.8 cm with DLP + and from 66.7 cm to 60.3 cm with DLP−, corresponding to mean variations of −6.4 cm and −8.7 cm, respectively (*P* < .001; Figure 5). Intragroup analyses confirmed that reductions were significant from D7 onwards on both sides (*P* < .001 for all timepoints), but the magnitude of edema resorption was consistently greater on the silicone-treated side for abdominal measurements. Clinical cases using the prototype are presented in Figures 6 and 7. An additional example of a bilateral DLP + garment is provided in Supplemental Figure.

### Patient-Reported Outcomes and Satisfaction

At the end of the study, all patients completed the standardized questionnaire (Supplemental Table). Consistent with the measurement findings, most patients perceived a benefit with the silicone-enhanced garment: A total of 28 out of 30 patients rated positively agreed or strongly agreed that the DLP technology improved skin quality, and all 30 patients agreed or strongly agreed that it accelerated the reduction of swelling, indicating a strong perceived postoperative benefit of the garment. In the questionnaire, this corresponded to high mean scores for skin quality (4.37 ± 0.93) and swelling reduction (4.63 ± 0.49) (Table 2). Regarding overall satisfaction, patients rated the DLP + side with a mean score of 4.50 ± 0.90. In contrast, the conventional DLP− side received a lower mean score of 3.00 ± 0.95, reflecting more neutral or negative evaluations. Comfort during use was also higher for the silicone side (mean 3.70 ± 0.95). No patient selected the lowest comfort category (“very uncomfortable”). Similarly, ease of use favored the DLP + garment, with mean scores of 3.88 ± 1.13 for inserting the garment and 4.25 ± 0.46 for removing it. Overall, patient-reported outcomes consistently demonstrated higher satisfaction, comfort, and perceived postoperative improvement with the silicone-enhanced DLP + side compared with the conventional compression garment.

**Table 2.** Questionnaire Scores

Question	Mean	SD
Q1_SkinQuality (“Do you think the DLP technology improves skin quality?”)	4.37	0.93
Q2_SwellingReduction (“Do you think the DLP technology accelerates the reduction of swelling?”)	4.63	0.49
Q3_BruisingResolution (“Do you think the DLP technology accelerates the disappearance of bruising (ecchymosis)?”)	5.00	0
Q4_SkinComfort (“Does the DLP technology leave the skin more comfortable?”)	3.93	1.08
Q5_SkinFirmness (“Does this technology leave the skin firmer?”)	4.33	1.12
Q6_HealingBetter (“Do you think DLP technology side has healed better postoperatively compared to the other side?”)	4.53	0.82
Q7_ComfortDuringUse (“During the use of DLP technology, did you feel comfortable”)	3.70	0.95
Q8_InsertUseEase (“How would you rate the use of the Panty insert?”)	3.88	1.13
Q9_InsertRemovalEase (“How would you rate the removal of the Panty insert with DLP technology?”)	4.25	0.46
Q10_OverallDLPPlus (“Your overall opinion of the DLP + side only regarding shape and contour.”)	4.50	0.90
Q11_OverallDLPMinus (“Your overall opinion of the DLP− side only regarding skin firmness and contour.”)	3.00	0.95

Ratings were provided on a 1-5 scale, where 1 represented the worst outcome and 5 represented the best outcome. DLP, Drain Lipo Panty.

## DISCUSSION

Persistent postoperative edema remains a major concern following procedures such as lipoabdominoplasty and lower limb liposuction, where disruption of lymphatic pathways is common. Despite the widespread use of compression garments, their design has changed little over the past decades, and robust evidence supporting their effectiveness in reducing lymphatic complications remains limited.^7-10^ Our study introduces and evaluates a novel compression garment, the Drain Lipo Panty (DLP), which integrates silicone strips aligned with known lymphatic pathways to promote dynamic, movement-dependent drainage. The rationale for this approach is supported by advances in lymphatic mapping techniques, particularly indocyanine green (ICG) lymphography. Studies by Suami et al^3^ and Bassalobre et al^5^ have demonstrated the complexity and plasticity of lymphatic drainage pathways in the lower limbs and abdomen, including rerouting toward alternative nodal basins following surgery. These findings suggest that uniform compression may be suboptimal and that targeted, anatomically informed compression could better support physiological drainage. The DLP's skin-adherent silicone strips are designed to provide gentle, intermittent mechanical stimulation, a concept akin to the micro-massage effect seen in kinesiology taping, which has shown benefit in breast cancer–related lymphedema.^11^ Unlike conventional garments that apply constant pressure, this dynamic compression may promote lymphangiomotor activity and facilitate interstitial fluid mobilization, particularly in the early postoperative phase when lymphatic transport is compromised.^12,13^

Beyond the biomechanical principles, patient compliance is essential for any compression therapy to be effective. Previous studies have identified garment-related discomfort, poor fit, and overheating as major barriers to adherence.^14-16^ In contrast, the DLP was well tolerated in our cohort, suggesting that the integration of silicone elements did not negatively impact wearability. On the contrary, patients reported enhanced comfort and satisfaction, an encouraging finding, given that compression therapy often fails due to poor tolerance. Although compression garments are routinely used in aesthetic surgery, there is a notable lack of randomized or comparative studies evaluating their true impact on postoperative outcomes.^7,17-21^ Some reports have even suggested that compression may exacerbate edema formation, particularly if not anatomically optimized.^22^ Our DLP design aims to address this gap by combining conventional elastic support with targeted lymphatic stimulation. This represents a conceptual shift from purely mechanical containment to biofunctional interaction.

While our intra-patient, crossover design offers certain methodological strengths, limitations remain. These include the absence of blinding, a modest sample size, and lack of validated quality-of-life instruments specific to lymphedema, such as the LyQLI or Lymph-ICF.^12^ Moreover, individual lymphatic anatomy was not mapped preoperatively, although this could be a future avenue for personalized garment fitting using ICG lymphography.^2,3^ The follow-up period in this study was limited to the early postoperative phase, and we therefore cannot determine whether the observed improvements translate into long-term benefits in terms of edema control, tissue remodeling, or aesthetic outcome. Additionally, while the intra-patient comparison reduces interindividual variability, liposuction volumes were not quantified separately for each side. Consequently, subtle differences in aspirate volume may have contributed to variations in circumference measurements, and this potential confounding factor should be acknowledged when interpreting the findings.

## CONCLUSIONS

This prospective study suggests that the Drain Lipo Panty (DLP), a novel compression garment integrating anatomically aligned silicone strips, may enhance postoperative recovery by promoting edema resorption and improving patient comfort after body contouring procedures. The skin-adherent silicone elements likely contribute to lymphatic drainage through dynamic, movement-dependent stimulation, offering a promising refinement to conventional garment design. Patients reported greater satisfaction, improved skin appearance, and reduced swelling on the DLP-treated side, though confirmation in larger, multicenter randomized trials is needed. Future studies may incorporate objective lymphatic imaging, such as ICG lymphography, to clarify mechanisms and outcomes. Additional prototypes are in development for other surgical regions, with the goal of extending the benefits of targeted lymphatic stimulation to a broader range of aesthetic and reconstructive procedures.

## Supplemental Material

This article contains supplemental material located online at https://doi.org/10.1093/asjof/ojag010.

## Supplementary Material

ojag010_Supplementary_Data
