# Supplementary material for: A Novel Compression Garment With a Dynamic External Lymphatic Drainage System: A Proof of Concept Study
Source: Aesthet Surg J Open Forum. 2026 Jan 28;8:ojag010. doi: 10.1093/asjof/ojag010 (PMC13098131; doi:10.1093/asjof/ojag010)
Supplement: ojag010_Supplementary_Data [file ojag010_Supplementary_Data.zip › Supplemental_Table.docx]

| Question | 1 | 2 | 3 | 4 | 5 |
| --- | --- | --- | --- | --- | --- |
| Do you think the DLP technology improves skin quality? | 1 = Strongly disagree | 2 = Disagree | 3 = Neutral | 4 = Agree | 5 = Strongly agree |
| Do you think the DLP technology accelerates the reduction of swelling? | 1 = Strongly disagree | 2 = Disagree | 3 = Neutral | 4 = Agree | 5 = Strongly agree |
| Do you think the DLP technology accelerates the disappearance of bruising (ecchymosis)? | 1 = Strongly disagree | 2 = Disagree | 3 = Neutral | 4 = Agree | 5 = Strongly agree |
| Does the DLP technology leave the skin more comfortable? | 1 = Strongly disagree | 2 = Disagree | 3 = Neutral | 4 = Agree | 5 = Strongly agree |
| Does this technology leave the skin firmer? | 1 = Strongly disagree | 2 = Disagree | 3 = Neutral | 4 = Agree | 5 = Strongly agree |
| Do you think DLP technology side has healed better postoperatively compared to the other side? | 1 = Strongly disagree | 2 = Disagree | 3 = Neutral | 4 = Agree | 5 = Strongly agree |
| During the use of DLP technology, did you feel: | 1 = Very uncomfortable | 2 = Uncomfortable | 3 = Neutral | 4 = Comfortable | 5 = Very comfortable |
| How would you rate the use of the Panty insert? | 1 = Very difficult | 2 = Difficult | 3 = Neutral | 4 = Easy | 5 = Very easy |
| How would you rate the removal of the Panty insert with DLP technology? | 1 = Very difficult | 2 = Difficult | 3 = Neutral | 4 = Easy | 5 = Very easy |
| Your overall opinion of the DLP+ side only regarding shape and contour. | 1 = Very poor | 2 = Poor | 3 = Fair | 4 = Good | 5 = Excellent |
| Your overall opinion of the DLP– side only regarding skin firmness and contour. | 1 = Very poor | 2 = Poor | 3 = Fair | 4 = Good | 5 = Excellent |
