# Supplementary figures and images for: A Novel Compression Garment With a Dynamic External Lymphatic Drainage System: A Proof of Concept Study
Source: Aesthet Surg J Open Forum. 2026 Jan 28;8:ojag010. doi: 10.1093/asjof/ojag010 (PMC13098131; doi:10.1093/asjof/ojag010)

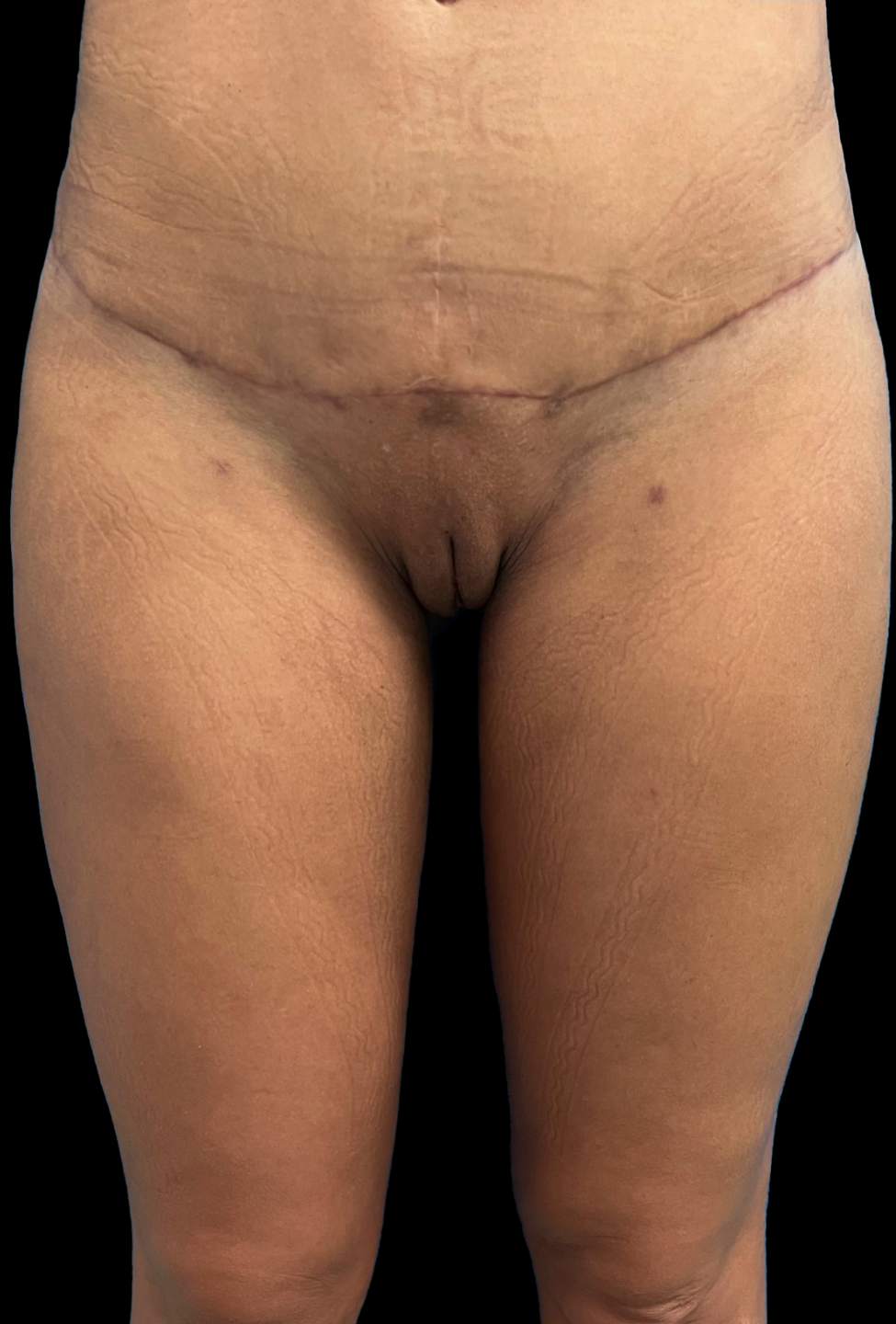

Supplement: ojag010_Supplementary_Data [file ojag010_Supplementary_Data.zip › Supplemental_Figure 1C.pdf]

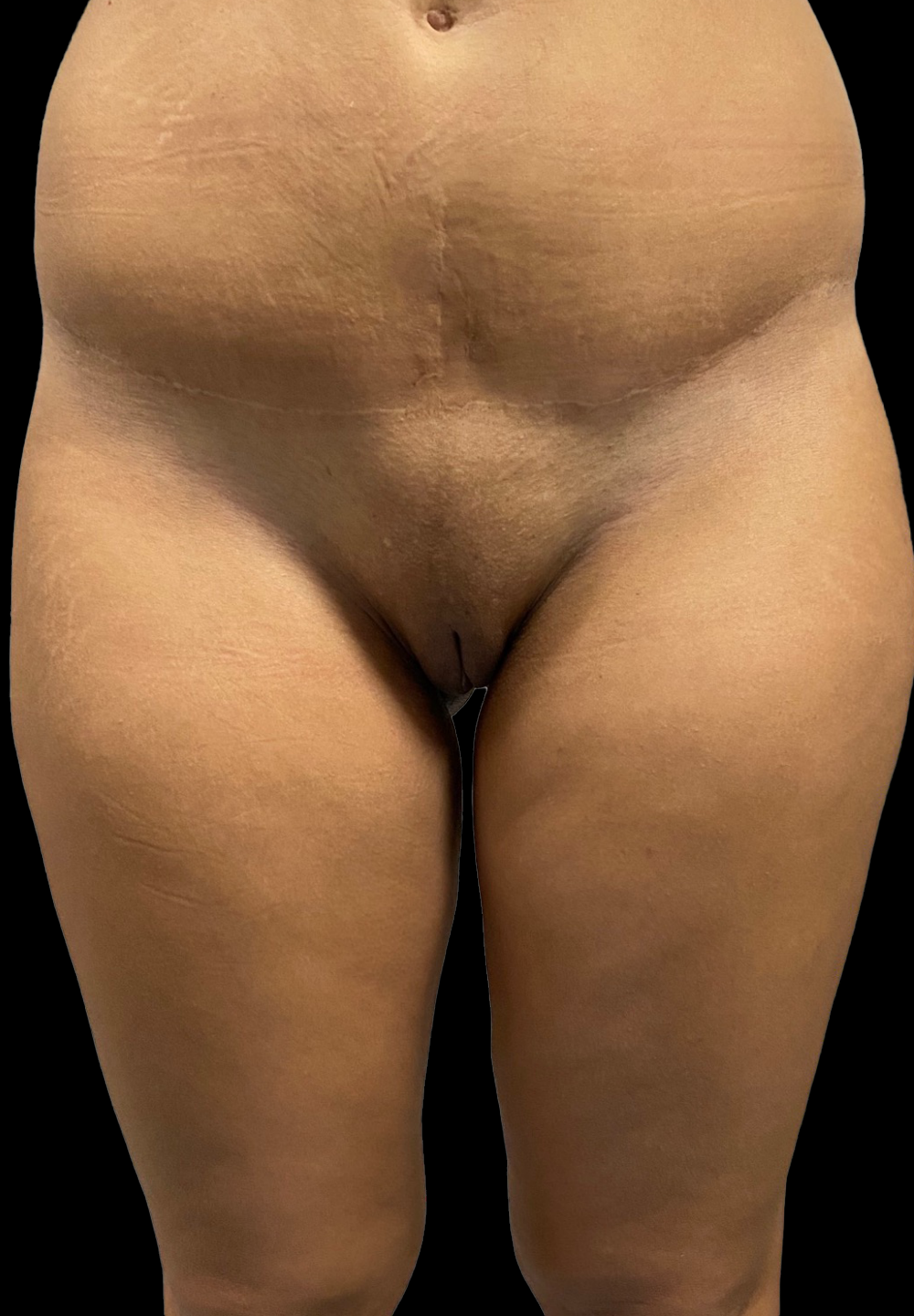

Supplement: ojag010_Supplementary_Data [file ojag010_Supplementary_Data.zip › Supplemental_Figure 1A.pdf]

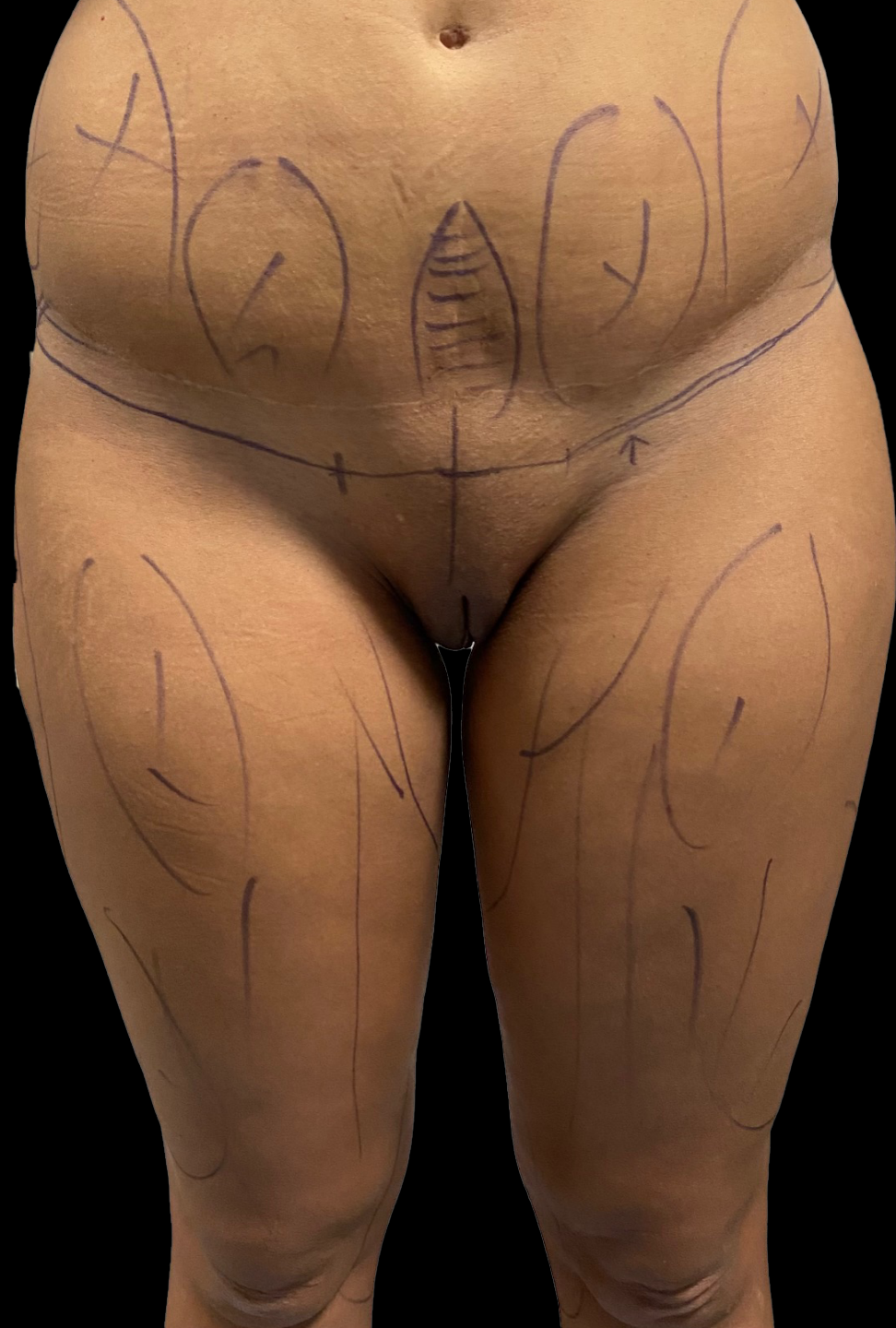

Supplement: ojag010_Supplementary_Data [file ojag010_Supplementary_Data.zip › Supplemental_Figure 1B.pdf]
